# Handgrip strength is associated with improved spirometry in adolescents

**DOI:** 10.1371/journal.pone.0194560

**Published:** 2018-04-11

**Authors:** Maia Phillips Smith, Marie Standl, Dietrich Berdel, Andrea von Berg, Carl-Peter Bauer, Tamara Schikowski, Sibylle Koletzko, Irina Lehmann, Ursula Krämer, Joachim Heinrich, Holger Schulz

**Affiliations:** 1 Institute of Epidemiology, Helmholtz Zentrum München–German Research Center for Environmental Health, Neuherberg, Germany; 2 Department of Public Health and Preventive Medicine, St. George’s University, Grenada, West Indies; 3 Research Institute, Department of Pediatrics, Marien-Hospital Wesel, Wesel, Germany; 4 Department of Pediatrics, Technical University of Munich, Munich, Germany; 5 IUF Leibniz Research Institute for Environmental Medicine and Medical Faculty, Heinrich Heine University of Düsseldorf, Düsseldorf, Germany; 6 Dr von Hauner Children’s Hospital, Ludwig Maximilians University of Munich, Munich, Germany; 7 Department Umweltimmunologie / Core Facility Studien Helmholtz-Zentrum für Umweltforschung–UFZ Leipzig, Germany; 8 Comprehensive Pneumology Center Munich (CPC-M), Member of the German Center for Lung Research, Munich, Germany; New York University School of Medicine, UNITED STATES

## Abstract

**Introduction:**

Pulmonary rehabilitation, including aerobic exercise and strength training, improves function, such as spirometric indices, in lung disease. However, we found spirometry did not correlate with physical activity (PA) in healthy adolescents (Smith ERJ: 42(4), 2016). To address whether muscle strength did, we measured these adolescents’ handgrip strength and correlated it with spirometry.

**Methods:**

In 1846 non-smoking, non-asthmatic Germans (age 15.2 years, 47% male), we modeled spirometric indices as functions of handgrip strength by linear regression in each sex, corrected for factors including age, height, and lean body mass.

**Results:**

Handgrip averaged 35.4 (SD 7.3) kg in boys, 26.6 (4.2) in girls. Spirometric volumes and flows increased linearly with handgrip. In boys each kg handgrip was associated with about 28 mL greater FEV1 and FVC; 60 mL/sec faster PEF; and 38 mL/sec faster FEF2575. Effects were 10–30% smaller in girls (all p<0.0001) and stable when Z-scores for spirometry and grip were modeled, after further correction for environment and/or other exposures, and consistent across stages of puberty.

**Conclusions:**

Grip strength was associated with spirometry in a cohort of healthy adolescents whose PA was not. Thus, research into PA’s relationship with lung function should consider strength as well as total PA. Strength training may benefit healthy lungs; interventions are needed to prove causality.

## Introduction

Physical activity (PA) has been reported to protect against chronic diseases, such as cardiovascular disease, obesity and insulin resistance, [1] across the lifespan. Pulmonary benefits appear to be heterogeneous: PA is part of pulmonary rehabilitation for lung disease,[2] and higher PA levels are associated with better functioning in lung disease[3, 4] but in healthy young people the association is not as well studied and sometimes appears to be subgroup-specific [5] [6] or nonexistent.[7] PA is most often quantified as time spent in moderate or vigorous activity [8] [9] with less priority given to muscle-building resistance training.[10] However, muscle strength may be independently important.

Muscle-building interventions as short as a few weeks can increase levels of growth hormone and testosterone [11] in healthy adults, and increase bone density in aging women [12]; and resistance training is a standard component of pulmonary rehabilitation. [13] One common marker of general muscle strength is handgrip, [14–16] and interventions which improve upper-body strength also improve grip [17] so handgrip is gaining popularity as a robust and low-cost indicator of muscle strength. [14–16, 18, 19] In addition to being a general marker of good health in children [18] and adults, [20] grip strength is associated with better spirometric lung function in some studies of healthy children[21] and adults[22]. However, the association is best studied and proven for the elderly [13, 23] and those with obstructive lung diseases such as COPD [13], cystic fibrosis[24] and asthma, [25] in which weaker grip cross-sectionally indicates both presence and severity.

It is plausible that the observed positive relationships between PA and spirometric indices [3–5] may be explained partly by muscle strength, rather than or in addition to total activity. Athletes’ aerobic performance is often limited by respiratory-muscle fatigue [26, 27] which can be improved by targeted muscle training: inhaling or exhaling against resistance strengthens muscles involved in respiration, such as the diaphragm, intercostals and abdominals. [28] This improves inspiratory and expiratory flow rates in both healthy [28]and diseased [29] populations. It is thus plausible that strengthening the thoracic muscles in other ways, such as through resistance training, may also provide pulmonary benefits in health, as it is known to do in disease.[2, 30, 31] Indeed, spirometric indices often improve following participation in sports that exercise the thorax, such as swimming [32] and yoga. [33] However, although respiratory-muscle strength and/or pulmonary function often correlate with grip strength [21, 23, 29] the association is not well studied: these studies tend to be small, and many do not correct for body size or shape and thus may be confounded by them. Pairwise comparisons of groups (e.g. athletes vs. non-athletes, boxers vs. rowers[6]) may be especially vulnerable to such confounding, especially if one group is strongly selected. Furthermore, handgrip is so reliably associated with health that reduced grip may indicate frailty in general, rather than muscle strength in particular, when comparing patients to non-patients. Lastly, the known benefits of targeted muscle training in specific populations do not necessarily translate to an association between spirometric indices and handgrip muscle strength in healthy adolescents.

Our goal in this study was to establish whether strength was associated with better lung function in a healthy cohort where physical activity was not, [7] and thus to establish the plausibility of strength as a direct driver of improved spirometry in healthy lungs. In models corrected for confounders not of primary interest and limited to apparently lung-healthy adolescents, we investigated the association between handgrip strength and spirometric indices.

## Methods

### Study population

We combined spirometry, physical examinations, and questionnaires from the 15-year followup of two German cohorts born between 1995 and 1999 and living in the regions of urban Munich and rural Wesel, GINIplus and LISAplus; in which we previously found no relationship between spirometry and accelerometric PA.[7] GINIplus and LISAplus were approved by local Ethics Committees (Bavarian General Medical Council, Medical Council of North Rhine-Westphalia) and by written informed consent from participating families (parents or guardians.) We do not have the approval of the ethics committee nor of the subjects to make the data publicly available, but they are available to researchers who obtain approval of the GINIplus and LISAplus study steering committees (contact: Dr. Marie Standl, marie.standl@helmholtz-muenchen.de or Dr. Holger Schulz, schulz@helmholtz-muenchen.de) and the ethics committees and acceptance of a data transfer agreement from the legal department of the Helmholtz Zentrum München.

GINIplus was initiated to investigate the role of infant feeding on allergy development. Newborns with family history of allergy (N = 2252) were randomized in almost equal numbers to either partially or extensively hydrolysed whey, extensively hydrolysed casein, or cow’s milk formula; the remaining 3739 (the observation arm) were given no intervention formula. The current study samples both study arms, correcting for formula only as a confounder not of primary interest. (See S1 Text) Of the total 5991, 3199 were recontacted at age 15, of which 1801 completed handgrip testing and spirometry. Further details on study design, formulas and followup have been previously published [7, 34]and are in S1 Text.

LISAplus is a population-based cohort in the regions of Munich, Wesel, and also in Bad Honnef and Leipzig. No intervention, nutritional or otherwise, was used. Of 1812 subjects recruited at birth in Munich and Wesel (handgrip was not tested in Bad Honnef or Leipzig), 1107 were followed up at age 15, of which 529 completed handgrip tests and spirometry.

In both studies, sociodemographic data (parental education, birthweight, breastfeeding, pre- and postnatal tobacco exposure to age 6, pubertal status, asthma, and smoking at age 15) were reported at the initial survey (age 4–6 months) and followups to 15 years. Height, weight, lean body mass (LBM), handgrip and spirometric indices were measured objectively during the physical examination at 15 years.

Of the 2330 subjects from GINIplus and LISAplus who completed handgrip and spirometry at age 15, 1846 completed LBM measures and confirmed no asthma [35]or smoking. These 1846 are our current study population. Of these, 1574 had complete data on spirometric confounders such as environmental exposures (e.g. PM_2.5_, and NO_x_), 1598 had data on puberty, and 987 had accelerometric PA measured, and were thus included in the sensitivity analyses correcting for these factors.

### Spirometric protocol

Spirometry was performed according to ATS/ERS recommendations, using a pneumotachograph-type spirometer (EasyOne Worldspirometer, ndd, Zurich, Switzerland) which has demonstrated volume accuracy of +/- 3% over at least four years with no significant nonlinearity. [36] Indices were taken from the maneuver with the largest sum of FEV1 and FVC[37]. A detailed protocol is given in S1 Text. Z-scores were calculated using reference values from the Global Lung Initiative 2012.[38] Because of the strong intercorrelation between spirometric indices within subject, we considered all for which GLI predicted values were available and compared their results. Of particular interest was the comparison between peak expiratory flow (PEF), the most effort-dependent flow, and forced expiratory flow between 25 and 75% of FVC (FEF2575) as the least. We also considered forced expiratory volume in 1 second (FEV1), forced vital capacity (FVC), and FEV1/FVC ratio as indicator of airflow limitation.

### Grip-strength protocol

Handgrip strength, quantified as the average of the better of two tests for each hand in accordance with the protocol in the PURE study[20] was measured using a validated TKK 5101 Grip D Dynamometer adjusted to fit the handspan of each subject, as detailed in the HELENA study. [18] For each hand subjects were instructed to let the arm hang free, squeeze the handle as hard as possible, and hold for two seconds; then release. Grip strength was measured to an accuracy of 0.1 kg. Z-scores for grip strength were calculated based on age from the HELENA study [18].

### Statistical methods

All analyses were conducted using Statistical Analysis System (SAS) 9.2 or 9.3. All analyses were stratified by sex. P-value for significance was 0.05. Each spirometric index was modeled as normally-distributed linear function of grip strength and other confounders.

Because most spirometric indices correlate with each other, many models will be similar. We present models of all four indices to allow intercomparison between them and with models presented in the literature.

Linearity of associations was confirmed for all models by visual comparison of the regression line to a locally-weighted curve (LOESS) fitted in SAS, which found no inflection points or indications of threshold or ceiling effects. Inspection of q-q plots confirmed normality for grip strength in each sex and for all spirometric indices except FEV1/FVC, which was slightly skewed: however, the associations we found were still close to linear.

To check for effect modification, several nested models were fit. These are described below: confounders are described and defined in S1 Text.

#### Basic model

Preliminary analyses showed that, in addition to age, sex and height, spirometry was strongly associated with body frame size indicated as weight, body mass index (BMI), or lean body mass (LBM) as measured by bioelectric impedance. LBM was chosen as the best single indicator. Thus all models are corrected for LBM in addition to cohort-specific effects (nutritional intervention and study center Munich vs. Wesel), as well as age and height for models which use raw values rather than Z-scores.

#### Correlates of lung function

This model additionally corrected for correlates of lung health that were included when we found spirometry was not associated with physical activity.[7] These were parental education, BMI, birthweight, breastfeeding duration, pre- and postnatal tobacco-smoke exposure and air pollution (annual exposure to PM_2.5_ and NO_x_.)

#### Puberty

Puberty is associated with upper-body strength and growth rate, so in those who provided data we corrected for 5-level pubertal category using a validated self-report scale [39] based on the well-known Tanner scale.

#### Physical activity

Although we had found no link between PA and spirometry in this cohort, to be on the safe side we corrected for mean daily minutes moderate-to-vigorous PA in those 987 subjects (53%) with accelerometry. Accelerometry recruitment, protocols, data handling, and findings have been previously published [34] and are in S1 Text.

#### Predicted values

Spirometry and grip strength are strongly associated with age and height [38, 40] and the relationship for spirometry is nonlinear [38] so we also modeled Z-scores for spirometry[38] as function of Z-scores for grip strength. [18, 40]

## Results

### Study population

The current study population of non-smoking, non-asthmatic adolescents was representative of the 15-year followup of GINIplus and LISAplus. (Table 1) Boys were likeliest to be mid- or late-pubertal (stages 3 and 4) while girls tended to be late- or postpubertal (stages 4 and 5.) Previous research [7] found the 15-year followups had less exposure to tobacco, were drawn from more highly educated and urban families, and were likelier to be female than those lost to followup: and their height, weight, and BMI fit a German reference. [41]

**Table 1:** Population characteristics.

	Boys	Girls
N	1846	
Male, N, %	867, 47	
Age at exam, years	15.2 (0.28)	15.2 (0.30)
Height, cm	176 (7.6)	167 (6.2)
Weight, kg	64.8 (12.8)	58.6 (9.8)
BMI, kg/m^2^	20.7 (3.3)	20.9 (3.0)
Lean body mass, kg	51.9 (8.1)	42.1 (5.4)
From Munich, %	57	55
Parents highly educated, %^1^	68	70
Valid spirometry, %	100	100
Valid grip-strength data, %	100	100
Pubertal stage,^2^ %		
1 (pre-pubertal)	0.4	0
2 (early pubertal)	4.8	0
3 (mid-pubertal)	37.7	4.6
4 (late pubertal)	55.7	78.7
5 (post-pubertal)	1.3	16.8
Spirometry		
FEV1, L	3.84 (0.65)	3.21 (0.43)
FVC, L	4.51 (0.76)	3.65 (0.51)
FEV1/FVC, %	85.3 (6.1)	88.4 (5.9)
PEF, L/sec	7.70 (1.3)	6.55 (0.95)
FEF2575, L/sec	4.13 (1.0)	3.75 (0.79)
Spirometry: Z-score^3^		
FEV1	-0.55 (0.97)	-0.52 (0.90)
FVC	-0.56 (0.95)	-0.47 (0.90)
FEV1/FVC	-0.05 (0.96)	-0.07 (0.99)
FEF2575	-0.43 (0.96)	-0.32 (0.93)
Grip strength^4^, kg	35.4 (7.2)	26.6 (4.1)
Grip strength^5^, Z-score	-0.58 (1.0)	0.01 (0.86)

1) Higher-educated parent entered university or higher.

2) Pubertal stage from validated self-report scale [39] based on the Tanner scale[42, 43].

3) From Global Lung Initiative, 2012.[39] Predicted values do not exist for PEF.

4) Grip measured as average of both hands, with each hand best of up to 2 trials.

5) Predicted values from HELENA study[18]

All measures given as % of those with data; centrally-distributed measures given as mean (SD) unless otherwise stated.

### Lung function

FEV1 and FVC averaged 3.84 (SD 0.65) and 4.51 (0.76) L for boys, 3.21 (0.43) and 3.65 (0.51) for girls. FEV1, FVC and FEF2575 were about half a standard deviation below predicted values in both sexes (GLI predicted values do not exist for PEF.) However, FEV1/FVC was close to predicted.

### Grip strength

Boys’ and girls’ grip strength averaged 35.4 (SD 7.2; range 16.6–64.5) and 26.6 (4.1; 14.7–42.4) kg; Z-scores [18] were -0.58 (1.0) and 0.01 (0.86).

### Spirometric indices and grip strength

**Fig 1:**
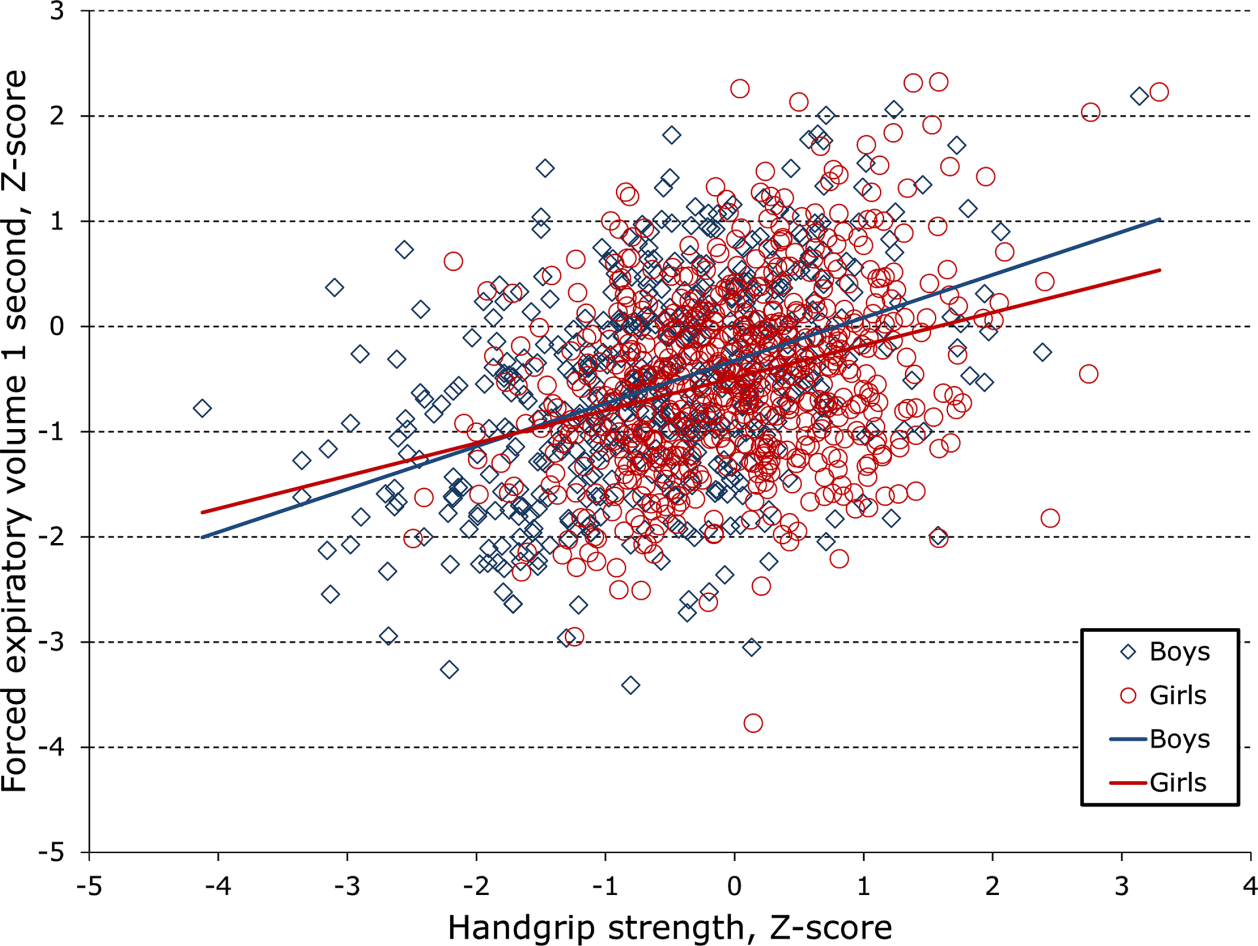
Handgrip strength is positively associated with lung volume in both male and female adolescents.

Spirometric volumes (FEV1, FVC) and flows (FEF2575, PEF) were significantly and linearly associated with grip strength (Table 2, Fig 1). After correction for the age of height and LBM, each additional kg of grip strength in boys was associated with 29 mL greater FEV1, and 28 mL greater FVC. For girls the corresponding numbers were 20 mL FEV1 and 21 mL FVC (all p<0.0001). Flows were also elevated with greater handgrip strength, with each kg of grip strength associated with 59 mL/sec greater PEF and 38 mL/sec greater FEF2575 in boys, 53 and 30 mL/sec in girls (all p<0.0001.) FEV1/FVC was also elevated in stronger adolescents, but the effect was only significant in boys (0.10%/kg, p = 0.02.)

**Table 2:** Spirometric indices and grip strength. **Basic Model.** Corrected only for age, height, lean body mass, nutritional intervention, and study center (Munich/Wesel).

		Grip strength, kg				% of variance explained by full model
		Slope estimate per kg	Std. error	P	% of variance explained by grip	
Boys	FEV1, mL	28.52	2.90	<0.0001	4.66	58.3
	FVC, mL	28.23	3.05	<0.0001	3.40	65.6
	FEV1/FVC, %	0.096	0.041	0.02	0.62	5.67
	PEF, mL/sec	59.49	7.25	<0.0001	5.01	35.6
	FEF2575, mL/sec	38.01	6.17	<0.0001	3.43	22.0
Girls	FEV1, mL	20.12	3.29	<0.0001	2.40	38.3
	FVC, mL	20.52	3.70	<0.0001	1.78	44.5
	FEV1/FVC, %	0.054	0.056	0.34	0.08	7.00
	PEF, mL/sec	52.68	8.44	<0.0001	3.36	17.1
	FEF2575, mL/sec	29.60	7.39	<0.0001	1.51	9.37

**Bold text** for index with most variance explained by grip for that sex.

**Fig 2:**
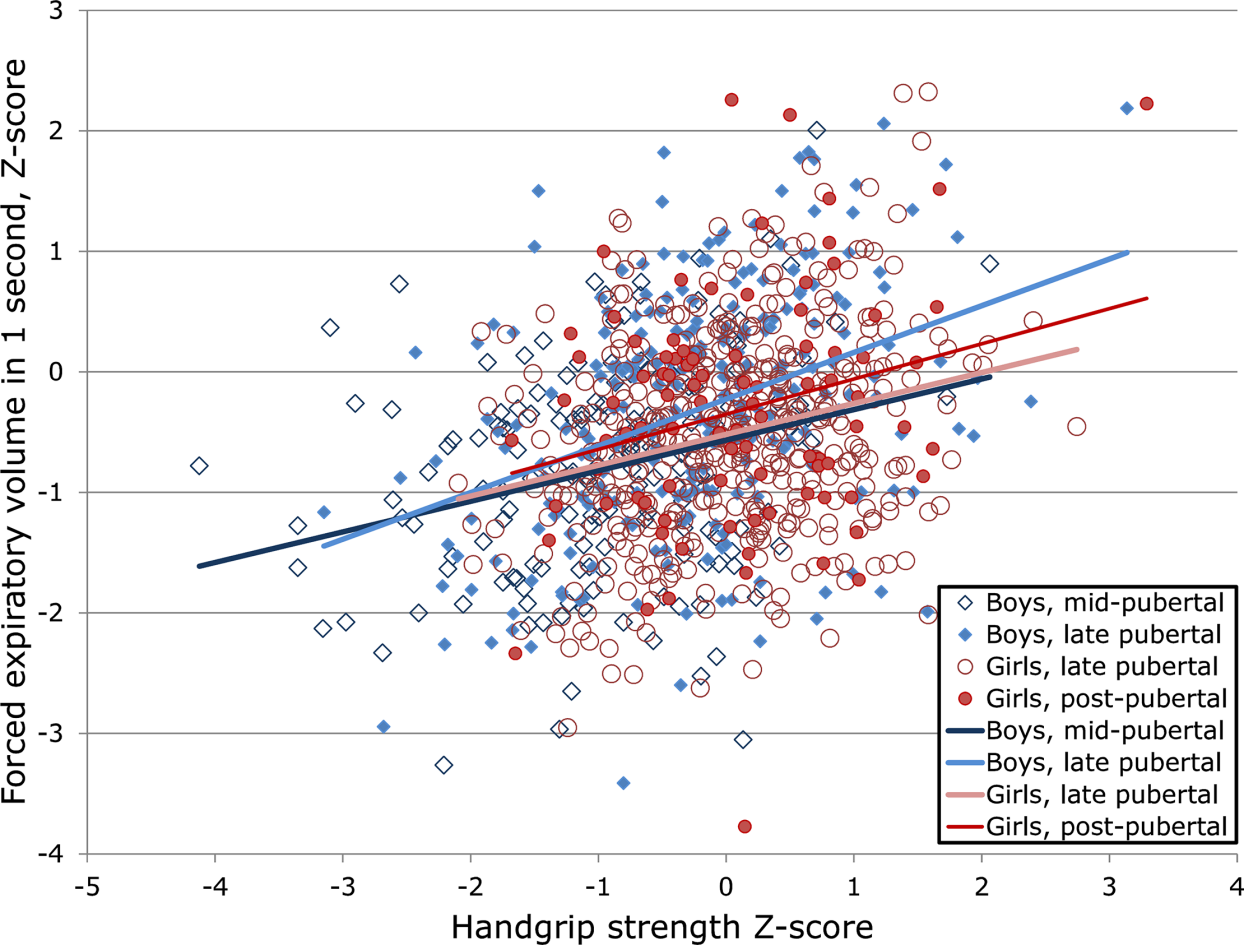
Handgrip strength is positively associated with lung volume in both male and female adolescents in different stages of puberty.

Effect sizes were only slightly smaller in the models corrected for BMI and/or weight in addition to LBM; correlates of lung function; puberty; or PA (Table 3). Effects for boys and girls were comparable across pubertal stages (Fig 2) The index with most variance explained by grip strength was usually FEV1, but occasionally PEF. Relationships between spirometry and grip strength (Fig 1) were close to linear even for the skewed FEV1/FVC. Models that did not consider any measure of body frame size (BMI, LBM, or weight) found effects about 30% larger (not shown.)

**Table 3:** Spirometric indices and grip strength, sensitivity analyses. All models corrected for age, height, lean body mass, nutritional intervention and study center (Munich/Wesel) as in Basic Model (Table 2).

		Grip strength, kg; mean of both hands														
		Lung health: Corrected for parental education, BMI, birthweight, breastfeeding duration, prenatal smoke, smoke at home up to age 6, and NO_x_, and PM_2.5_ at age 15^1^ N = 1572					Puberty: Corrected for pubertal status^2^ N = 1598					Physical activity: Corrected for daily minutes moderate-to-vigorous physical activity N = 987^3^				
		Slope estimate per kg	Std. error	P	% variance explained		Slope estimate per kg	Std. error	P	% variance explained		Slope estimate per kg	Std. error	P	% of variance explained	
					Grip	Full model				Grip	Full model				Grip	Full model
Boys	FEV1, mL	**27.01**	**3.28**	**<0.0001**	**3.92**	**58.0**	**26.92**	**3.29**	**<0.0001**	**3.71**	**59.4**	**25.95**	**4.11**	**<0.0001**	**3.85**	**58.8**
	FVC, mL	27.42	3.42	<0.0001	3.00	66.1	26.21	3.42	<0.0001	2.66	66.7	26.14	4.29	<0.0001	3.04	65.1
	FEV1/FVC, %	0.081	0.045	0.08	0.40	7.93	0.095	0.046	0.04	0.54	6.33	0.076	0.056	0.17	0.41	6.66
	PEF, mL/sec	54.24	8.13	<0.0001	3.91	36.3	53.64	8.22	<0.0001	3.68	36.9	49.49	10.1	<0.0001	3.46	38.2
	FEF2575, mL/sec	36.66	6.97	<0.0001	2.97	22.3	35.46	7.04	<0.0001	2.67	23.0	32.59	8.76	<0.0001	2.45	24.5
Girls	FEV1, mL	21.64	3.63	<0.0001	2.66	41.0	16.63	3.54	<0.0001	1.58	39.6	**23.03**	**4.29**	**<0.0001**	**3.21**	**40.0**
	FVC, mL	21.79	4.05	<0.0001	1.90	48.2	17.45	3.99	<0.0001	1.24	45.5	24.33	4.94	<0.0001	2.52	43.9
	FEV1/FVC, %	0.065	0.062	0.30	0.13	7.74	0.026	0.062	0.67	0.02	8.35	0.047	0.073	0.52	0.071	7.93
	PEF, mL/sec	**58.05**	**9.26**	**<0.0001**	**3.97**	**20.2**	**53.01**	**9.22**	**<0.0001**	**3.24**	**17.7**	45.52	11.4	<0.0001	2.50	16.3
	FEF2575, mL/sec	36.49	8.11	<0.0001	2.30	10.2	22.98	8.11	0.005	0.86	10.4	30.92	9.37	0.001	1.78	12.3

1) For full definitions and choice of confounders, see S1 Text and [7].

2) Pubertal development scores range from 1 (pre-pubertal) to 5 (post-pubertal.) [39]

3) Physical activity in the full accelerometry cohort profiled in [34]; in the spirometry cohort, [7]

**Bold text** for index with most variance explained by grip for that sex.

When spirometric Z-scores were modeled (Table 4) grip strength Z-score explained about 6% of the remaining variance in boys’ FEV1 and FVC, 2.5% of variance in boys’ FEF2575, and half that for girls (3.5 and 1.4%, respectively).

**Table 4:** Spirometric indices and grip strength, Z-scores. Corrected for lean body mass, nutritional intervention and study center (Munich/Wesel) as in Basic Model (Table 2.) Age and height are already corrected for in one or both Z-scores.

		Grip strength Z-score^2^				
		Slope estimate per Z-score unit	Std. error	P	% variance explained	
	Z-score^1^				Grip	Full model
Boys	**FEV1**	**307.5**	**48.2**	**<0.0001**	**6.21**	**21.6**
	FVC	289.5	46.1	<0.0001	5.88	23.3
	FEV1/FVC	62.7	51.6	0.23	0.27	4.46
	FEF2575	192.7	51.1	0.0002	2.51	9.39
Girls	**FEV1**	**246.8**	**48.4**	**<0.0001**	**3.69**	**10.7**
	FVC	235.4	48.9	<0.0001	3.21	12.7
	FEV1/FVC	9.99	54.8	0.86	<0.01	6.0
	FEF2575	151.0	50.4	0.003	1.35	5.33

1) Spirometric Z-scores from Global Lung Initiative 2012, [38].

2) Predicted values for grip strength from the HELENA study [18]

**Bold text** for index with most variance in spirometry explained by grip for that sex.

## Discussion

We found that grip strength was positively associated with lung volumes and flows, corresponding to 250–300 mL FEV1 per standard deviation of handgrip strength. Since research often uses handgrip to indicate general muscle strength,[14–16] and interventions which improve upper-body strength can also improve grip [17] and/or spirometry [6] [32] [44] [33] it is plausible that both handgrip and spirometric indices can be improved by exercises which strengthen the upper-body musculature. Further support is given to this by the close correlations between handgrip and the strength of the respiratory muscles. [45] While interventions are needed to prove causality, we suggest that strength training be added to the physical-activity recommendations for lung-healthy populations [10] as it has been to those with lung disease. [2, 13]

Our study population is among the first in which physical activity and handgrip strength were considered as independent correlates of spirometry, and thus we were able to establish the plausibility of various causal links. Since spirometry was associated with handgrip but not PA, [7] and PA did not significantly modify the association between spirometry and handgrip, total PA cannot be mediating the association between spirometry and handgrip in this population. Similarly, Greutmann et al [45] found that although 40 young heart-disease patients reported they were as physically active as healthy controls, they still had weaker handgrip and respiratory muscles, which suggests that their physical activity was the wrong type, or insufficiently intense, to build those muscles. It thus appears that total physical activity may have different correlates from strength-building specifically: indeed, previous research with a subset of our population [46] found that muscle-building exercise made up a relatively small fraction of total sporting activity, compared to aerobic team sports such as jogging and football. To avoid underestimating the benefits of physical activity, research into its correlates should consider the type of activity, or include measures of strength.[34, 46]

Our estimated association between spirometry and upper-body strength is consistent with earlier research on athletes whose sport strengthened the upper body: FEV1 was higher for athletes than nonathletes [6] [32], with effect sizes ranging from 14% (Vedala et al [44] to 30% (Yadav et al[33]). Our association is somewhat smaller than this, but our study is both larger and less confounded: we corrected not only for height but also for body proportions indicated by LBM. Residual confounding is still possible, since LBM includes leg muscles as well as chest: however, we found that adding LBM to a model of spirometry which considered height only decreased the association with grip by about 30%. Thus unmeasured variations in body proportions within this homogeneous population (ethnic Germans ages 14–17) would have to be quite large and nearly constant across pubertal stages to completely eliminate the remaining association. The same is true for other confounders, such as pollution exposure.

Our study objectively measures the correlates of interest (handgrip strength and spirometric indices) and the observed association was not confounded by known correlates of strength and lung function. However, selection occurred from initial recruitment (ethnic Germans) to followup at age 15; and completion of questionnaires and physical exam. Likewise, age range was narrow: our effects may be specific to young subjects, whose lungs have not fully matured and alveolarised. However, lung aging appears to be slower in active adults [47] suggesting that healthy aging lungs also benefit from PA. Lastly, we measured some environmental exposures over a longer period than others and thus some confounding may remain.[25]

## Conclusion

We show that muscle strength (here quantified as handgrip) was associated with better lung function even in healthy young people without known lung disease. Previous research with this same population showed that the association was not driven by general physical activity, suggesting that the correlates of strength (and therefore, perhaps also the results of strength training) differ from those of aerobic physical activity. While interventional data are needed to establish causation, clinical recommendations and PA interventions should consider including a strength-training component. Furthermore, we suggest that research into the association between PA and spirometry either explicitly consider the type of PA or also consider measures of strength, such as handgrip, as indicators of physical condition.

More generally, we find that the value of grip-strength testing is not limited to elderly and aging populations, but is also present in this cohort of healthy adolescents. Handgrip may serve as a low-cost, objective and safe indicator of physical condition for researchers and clinicians.

## Supporting information

S1 Fig Recruitment for GINIplus and LISAplus cohorts from birth to the current study and sensitivity analyses. (TIF)

S1 Text Details on recruitment of GINIplus and LISAplus, exclusion criteria for the current study, statistical models, choice of confounders, and protocols for grip strength and spirometry. (DOC)
