# Supplementary figures and images for: Handgrip strength is associated with improved spirometry in adolescents
Source: PLoS One. 2018 Apr 11;13(4):e0194560. doi: 10.1371/journal.pone.0194560 (PMC5894972; doi:10.1371/journal.pone.0194560)

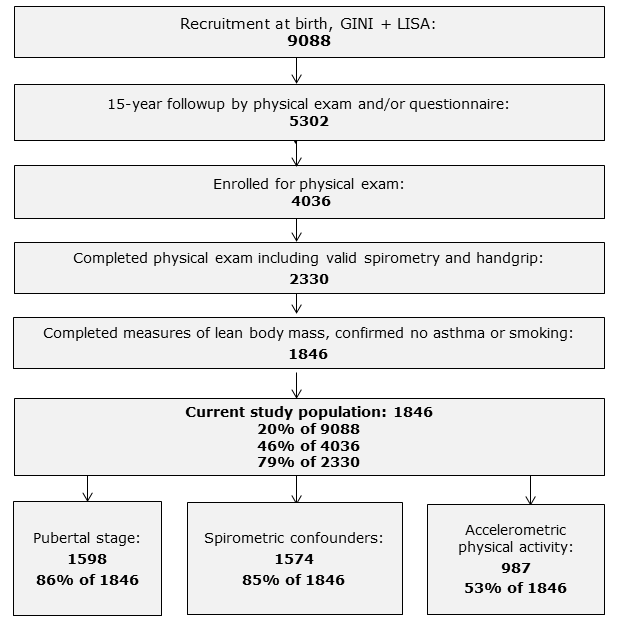

Supplement: S1 Fig — (TIF) [file pone.0194560.s001.tif]
