# Supplementary material for: Handgrip strength is associated with improved spirometry in adolescents
Source: PLoS One. 2018 Apr 11;13(4):e0194560. doi: 10.1371/journal.pone.0194560 (PMC5894972; doi:10.1371/journal.pone.0194560)
Supplement: S1 Text — (DOC) [file pone.0194560.s002.doc]

# Population Characteristics

## Recruitment and followup: GINIplus

A detailed overview of the recruitment protocol for GINIPlus (“German Infant Study on the influence of Nutrition Intervention (Plus environmental and genetic influences) on allergy development”) is available at the study’s website [1] and has been published previously. [2, 3, 4] GINIplus is a population-based prospective birth cohort consisting of two arms: one interventional and one observational. GINIPlus15 is the 15-year followup.

5991 healthy, full-term newborns were recruited between September 1995 and June 1998 (n=2949 in Munich, n=3042 in Wesel). Those with at least one parent or biological sibling with allergic disease were recruited for the nutritional intervention. 3739 unselected infants were recruited for the observational arm and given no formula (Figure 1a, Appendix 1, [5]). The intervention was a randomized, double-blind controlled trial of feeding with one of 3 hydrolysed formulas (partially or completely hydrolysed whey, or extensively hydrolysed casein: pHF-W, CHF-W, or eHF-C) versus cow’s milk formula (CMF) during first 4 months of life (n=1165 in Munich, n=1087 in Wesel). Details on randomization and blinding have been previously published.[3, 4, 6, 7] Following current recommendations, breastfeeding was encouraged for all families including those children enrolled in the intervention arm.

Followups included physical examinations and personal interviews at 1, 4, 8, 12 , 24 and 36 months, and at ages 6, 10 and 15; and questionnaires filled out by the child (age 10 and 15), the parents or both. For a detailed followup schedule see the website and previous publications. [2, 3, 4, 5] A flowchart for the 15-year followup has been previously published.[5]

Attempts were made to contact each family by postal mail and telephone when the subject was 15 years old. [5] Of the 5991 infants recruited for GINIplus, 3199 were successfully followed up by questionnaire and/or physical examination at age 15. [5] Of these, 1801 completed handgrip and spirometry; of these, 1416 denied asthma and smoking. Of these 1416, 48% received any nutritional intervention compared with only 38% of the original cohort. Since the intervention arm was restricted to children with a family history of allergic disease, this may represent ascertainment bias, greater health-consciousness, healthcare utilization or all of these. However, the four study formulas were present in roughly equal numbers in our subsample: the largest difference was for partially hydrolysed whey in girls. 27.0% of the study population that was given an intervention got pHF-W, compared with 24.7% of all GINIplus intervention subjects. This formula was not associated with asthma development [3] and thus differential exclusion due to asthma did not take place. We find that while our sample oversamples the intervention arm of GINIplus, there was no bias toward any specific formula.

## Recruitment and followup: LISAplus

Recruitment and followup protocols for LISAplus (Lifestyle-Immune System-Allergy: Influence of life-style factors on the development of the immune system and allergies in East and West Germany (Plus the influence of traffic emissions and genetics)) are available at the study’s website [8] and have been published previously. [5, 9, 10, 11] LISAplus is a prospective birth cohort in 4 regions of the former East and West Germany (Munich, Leipzig, Wesel, and Bad Honnef) instigated to examine the relationships between immune functioning and environmental and lifestyle exposures throughout life. No intervention, nutritional or other, was used in LISAplus.

3097 healthy, full-term newborns were recruited between November 1997 and January 1999 (n= 1467 from Munich, 976 from Leipzig, 348 from Wesel, and 306 from Bad Honnef) of which 1534 (50%) were followed up at age 15; of these 956 were from Munich (797) and Wesel (159). The current study samples only these children, since handgrip was not offered to the study centers in Bad Honnef and Leipzig.

Followups took place regularly on a similar schedule to that for GINIplus; for details see the website and previous publications. [9, 10, 11] Questionnaires were given monthly during the first year of life, every 6 months until age 2 years and thereafter at age 4, 6, 10 and 15 years, while medical examinations took place at ages 2, 6, 10 and 15.

A detailed comparison of the 15-year followup to the population at birth has been previously published. [5] While there was no strong bias towards preferential dropout in our cohort compared to the rest of the 15-year followup, the 15-year followup differed from the initial cohort with respect to contribution from Munich, education of the parents, and smoke exposure by the mother during and after pregnancy suggesting differential loss to followup.

## Exclusion Criteria:

The current study investigates the relationships between strength and healthy lung function, so we excluded children with asthma, smoking, or cystic fibrosis as well as those with missing data on spirometry or handgrip. Table S1 compares the study population to the full 15-year followup.

*Asthma:* Asthma was defined as in the GA2LEN study, namely as at least two of the following: self-reported asthma medication at age 15, self-reported wheezing in the past year, or diagnosed asthma ever reported by child or parent. [12, 13]

*Smoking:* Smoking was defined as self-reporting any tobacco smoking ever.

| **Table S1: Selection** | | | | | | |
| --- | --- | --- | --- | --- | --- | --- |
|  | Entire 15-year followup living in Munich and Wesel | | Study population  Completed handgrip and spirometry, confirmed no asthma or smoking | | P for selection if <0.05 | |
|  | Boys | Girls | Boys | Girls | Boys | Girls |
| N | 4306 | | 1846 | | -- | |
| Male | 51 | | 47 | | <0.0001 | |
| Age at exam | 15.3 (0.31) | 15.3 (0.32) | 15.2 (0.28) | 15.2 (0.30) | <0.0001 | 0.002 |
| Height, cm | 176 (7.5) | 167 (6.3) | 176 (7.6) | 167 (6.2) | -- | 0.04 |
| Weight, kg | 65.0 (13) | 58.8 (10) | 64.8 (12.8) | 58.6 (9.8) | -- | -- |
| BMI, kg/m^2^ | 20.8 (3.4) | 21.0 (3.1) | 20.7 (3.3) | 20.9 (3.0) | -- | 0.05 |
| Lean body mass, kg | 51.9 (8.2) | 42.0 (5.5) | 51.9 (8.1) | 42.1 (5.4) | -- | -- |
| From Munich | 59 | 59 | 57 | 55 | -- | 0.0007 |
| Parents highly educated^1^ | 65 | 69 | 68 | 70 | 0.03 | -- |
| Nutritional intervention^2^ | 32 | 32 | 37 | 37 | 0.0001 | <0.0001 |
| Breastfeeding: p for global null |  |  |  |  | -- | 0.005 |
| Never | 41 | 39 | 40 | 36 | -- | -- |
| 1-4 months | 10 | 11 | 10 | 10 | -- | -- |
| 5 months or more | 48 | 50 | 51 | 54 | -- | -- |
| PM2.5^3^ | 15.1 (2.2) | 15.1 (2.2) | 15.0 (2.2) | 15.2 (2.2) | -- | -- |
| NOx^3^ | 33.9 (8.9) | 33.8 (8.0) | 33.9 (8.7) | 33.7 (7.5) | -- | -- |
| Prenatal tobacco exposure | 12 | 13 | 11 | 11 | -- | -- |
| Postnatal tobacco exposure | 39 | 37 | 35 | 35 | 0.0008 | -- |
|  |  |  |  |  |  |  |
| Valid spirometry | 52 | 57 | 100 | 100 | ** | ** |
| Valid grip-strength data | 54 | 59 | 100 | 100 | ** | ** |
|  |  |  |  |  |  |  |
| **Spirometry** |  |  |  |  |  |  |
| FEV1, L | 3.83 (0.65) | 3.21 (0.42) | 3.84 (0.65) | 3.21 (0.43) | -- | -- |
| FVC, L | 4.51 (0.75) | 3.64 (0.50) | 4.51 (0.76) | 3.65 (0.51) | -- | -- |
| FEV1/FVC, % | 84.9 (6.4) | 88.2 (6.0) | 85.3 (6.1) | 88.4 (5.9) | 0.005 | -- |
| PEF, L/sec | 7.69 (1.3) | 6.55 (0.95) | 7.70 (1.3) | 6.55 (0.95) | -- | -- |
| FEF2575, L/sec | 4.09 (1.0) | 3.74 (0.80) | 4.13 (1.0) | 3.75 (0.79) | 0.01 | 0.04 |
| **Spirometry: percent predicted**^3^ |  |  |  |  |  |  |
| FEV1 | 92.9 (12) | 93.9 (10) | 93.2 (12) | 93.9 (11) | -- | -- |
| FVC | 93.6 (11) | 94.6 (11) | 93.6 (11) | 94.5 (11) | -- | -- |
| FEV1/FVC | 98.8 (7.5) | 98.8 (6.7) | 99.2 (7.0) | 99.0 (6.6) | 0.003 | -- |
| FEF2575 | 90.4 (21) | 93.3 (19) | 91.5 (21) | 93.7 (19) | 0.004 | -- |
|  |  |  |  |  |  |  |
| Grip strength^4^, kg | 35.4 (7.3) | 26.6 (4.2) | 35.4 (7.2) | 26.6 (4.1) | -- | -- |
| Asthma^5^ | 7.3 | 5.5 | 0 | 0 | ** | ** |
| Smoking | 4.7 | 4.7 | 0 | 0 | ** | ** |
| Missing data on asthma or smoking | 50 | 46 | 0 | 0 | ** | ** |
|  |  |  |  |  |  |  |
| 1) Higher-educated parent entered university or higher.  2) Nutritional intervention used only in intervention arm of GINIplus; here defined as 1 if any intervention formula was given and 0 if none. For details on selection and confounders see[4] (GINIplus) and [9] (LISAplus).  3) Average annual exposure at subject’s home address, age 15  Predicted values from Global Lung Initiative, 2012.[14] Predicted values do not exist for PEF.  4) Grip measured as average of both hands, with each hand best of up to 2 trials.  5) Asthma defined as in the GA2LEN study[13], as at least two of the following: doctor diagnosis since age 3, current asthma medicine, wheezing in the past year.  Binary measures given as % of those with data; centrally-distributed measures given as mean (SD) unless otherwise stated.  P-values from Wilcoxon’s two-tailed rank-sum test for binary and continuous variables, Kruskal-Wallis for categorical.  -- if p>0.05, ** if characteristic was used for inclusion and thus is defined as 0 or 100% in study population. | | | | | | |

# Statistical Methods:

All analyses were stratified by sex. All analyses used SAS 9.2. Populations were compared using nonparametric methods (detailed in table footer) since many variables were categorical or skewed. Spirometric indices were modeled as normally-distributed linear functions of grip strength and other confounders.

Indices considered were FEV1 and FVC as indicators of lung volume, PEF and FEF2575 as measures of expiratory muscle strength, and FEV1/FVC as indicator of airflow limitation. FEV1 and FVC were modelled as mL, FEV1/FVC as %, and FEF2575 and PEF as mL/sec. Z-scores were multiplied by 1000 before modelling.

Linearity of associations was confirmed for all models by visual comparison of the regression line to a locally-weighted curve (LOESS) also fitted in SAS, which found no inflection points or indications of threshold or ceiling effects. Inspection of q-q plots confirmed normality for grip strength in each sex and for all indices except FEV1/FVC, which was slightly skewed (mean 0.87, range 0.62-1.0). Rather than attempt to transform it to normality, we present associations as observed and caution that p-values may be slightly unreliable.

To check for effect modification, several nested models were fit. These are described briefly in the main text, and in more detail here.

# Models:

### Basic Model:

Preliminary analyses showed that, in addition to age, sex and height, spirometry was strongly associated with body frame size indicated as weight, BMI, or LBM measured by bioelectric impedance. LBM was chosen as the best single indicator: however the estimated relationship of spirometry with grip strength was very similar when other measures of frame size, such as BMI or total body weight, were used.. Thus all models are corrected for LBM in addition to cohort-specific effects (nutritional intervention and study center Munich vs. Wesel), as well as age and height for models which use raw values rather than Z-scores.

### Lung Health

Correlates of lung health were socioeconomic status as quantified by parental education (whether or not the better-educated parent entered university), BMI, birthweight, breastfeeding duration (never, first four months, or past four months) pre- and postnatal tobacco-smoke exposure (any vs. none, for each) and air pollution (annual average exposure to PM2.5 and NOx at the subject’s home address at age 15.)

### Puberty

Because of the association between upper-body strength, growth rate, and puberty, in the subset of subjects (1598/1846, 87%) who provided data we corrected for it.

### Physical Activity

Although physical activity is not associated with spirometric indices in this cohort,[5] we corrected for it (mean daily minutes moderate-to-vigorous physical activity, averaged over one week of accelerometer weartime) in a sensitivity analysis for those 987 subjects who provided accelerometric data (53%).

Triaxial accelerometers (ActiGraph GT3X, Pensacola, Florida) were worn on the dominant hip for 7 consecutive days, after which they were returned by mail. Device weartime was identified both by visual inspection of accelerometer tracings and by comparing the diary data to the results from the monitor according to the NWT algorithm of Troiano et al [[27](http://journals.plos.org/plosone/article?id=10.1371/journal.pone.0152217#pone.0152217.ref027)] using SAS programs published by NHANES.[15] Accelerometric data were processed into activity intensities minute by minute using the algorithm of Freedson et al.[16] Valid weekdays and weekend days had at least 7 or 10 hours recording, respectively; valid subjects had at least 3 valid weekdays and one valid weekend day.

Detailed accelerometry protocol is given elsewhere. [17]

### Predicted Values

Both spirometry and grip strength are strongly and positively associated with age and height, and the relationship for spirometry is nonlinear. [18] [14] To ensure that these effects did not drive results, we calculated predicted values for spirometry and grip strength and compared those results to those which considered raw values. Spirometric Z-scores were calculated based on Global Lung Initiative reference values.[14] Grip strength was modeled as percent predicted from European reference values. [18]

# Confounders:

*Height:* Height was measured using a calibrated “Körperhöhenmessgerät Dr Keller I” to a precision of 1 mm, without shoes or thick socks.

*Weight:* Weight was measured to a precision of 100 g using a calibrated device. Participants wore light clothing without shoes or thick socks.

*Lean body mass (LBM):* As the best indicator of body-frame size, we included lean body mass (LBM) in all models. LBM was measured with biometrical impedance analysis at the 15-year physical examination, using the Nutribox apparatus and Nutriplus software from Data Input GmbH (Darmstadt, Germany). Data were analysed according to the three-compartment model of body composition (fat, body cell mass, and extracellular mass.)

*Body mass index (BMI):* As an alternate measure of body size that almost all clinicians have access to, BMI was calculated from height and weight (measured objectively at the physical exam) as kg/m^2^.

*Birthweight****:*** Both cohorts were limited to full-term births and LISAplus specifically excluded subjects with low birthweight; however, some small effect may remain and was corrected for.

*Exclusive breastfeeding:* Exclusive breastfeeding was modelled as a three-level categorical predictor: never, between ages 1 and 4 months only, and to the fifth month or later, as reported by the mother.

*Prenatal smoke exposure:* We defined prenatal tobacco-smoke exposure as whether the mother reported smoking any cigarettes during pregnancy.

*Childhood secondhand-smoke exposure:* We defined childhood exposure to secondhand smoke as whether anyone in the household smoked up to the child’s age of 6.

*Air pollution:* Most subjects were also enrolled in the ESCAPE project, a multicentre study of air pollution exposure and childhood asthma prevalence. For project details see[10, 12, 19]. Air pollution was quantified as the annual average exposure to PM_2.5_ and NO_x_ at the subject’s home address at age 15. Baseline concentrations (mean (median); 5^th^, 95^th^ percentile) were 15.1 (14.3) 12, 18 µg/m^3^ for PM2.5, and 33.8 (32.6); 24, 47 µg/m^3^ for NOx. For further details on data collection and definitions, see [10, 12, 19].

*Puberty:* Puberty was categorized according to a validated self-rating scale [20] based on the well-known Tanner scale. [21, 22] Puberty ranged from 1 (prepubertal) to 5 (postpubertal), and was treated as a 5-level categorical variable with 3, “mid-pubertal” as reference for both sexes.

# Spirometric Protocol:

Spirometry was performed at the 15-year followup physical examinations for GINIplus and LISAplus. Measurements were performed in line with ATS/ERS recommendations[23] using a pneumotachograph-type spirometer (EasyOne Worldspirometer, ndd, Zurich, Switzerland), calibrated daily before spirometry with a 3-L calibration pump supplied by the manufacturer. , This device has demonstrated volume accuracy of +/- 3% over at least four years with no significant nonlinearity. [36]

Subjects were seated while wearing nose clips. They performed at least three but not more than eight trials per test under the guidance of trained and experienced examiners in order to obtain optimal flow-volume curves. Both flow-volume and volume-time curves were monitored by the examiner and visible to the participant to enable guided support of the participant.

Based on ATS/ERS acceptability criteria[23] and as recommended by [24] all tests were visually inspected by physicians to exclude manoeuvres performed incorrectly or with artefacts. Spirometric indices were taken from the best manoeuvre with the largest sum of FEV1 and FVC. Indices considered in the current study were FEV1; FVC, the ratio of FEV1 and FVC (FEV1/FVC), peak expiratory flow (PEF), and the mean flow rate between 25 and 75% of FVC (FEF2575). Indices were modelled both as raw values and as Z-scores based on Global Lung Initiative (GLI) reference values[14]when available.

In total, 2878 subjects from GINIplus and LISAplus underwent spirometry, of whom 2757 (96%) passed quality control

# Grip-strength Protocol:

Handgrip was measured at the 15-year followup physical exam for GINIplus and LISAplus, using a validated digital TKK 5101 Grip D Dynamometer (Takei, Tokyo, Japan.) For each hand subjects were instructed to let the arm hang free, squeeze the handle as hard as possible, and hold for two seconds; then release. Grip strength was measured to an accuracy of 0.1 kg. Strength of each hand was indicated by maximum of two trials; mean strength was the average of the maximum measurement for each hand, in accordance with the protocol in the PURE study.[25]

Z-scores for grip strength based on age and sex were calculated from the HELENA study. [18] In a sensitivity analysis (not shown) similar results were obtained using an alternate set of prediction equations based on age, sex, height and weight.[26]

**References:**
